# A Systematic Review of Substance Misuse Treatment Processes and Outcomes as Implemented in Prisons for Men in the UK

**DOI:** 10.1002/cbm.70008

**Published:** 2025-08-16

**Authors:** Kim Barnett, Noor Butt, Rosie Allen, Pauline Goodlad, Anne Krayer, Adam O'Neill, Peter Huxley, Catherine Robinson, Emily Peckham, Rob Poole

**Affiliations:** ^1^ Centre for Mental Health and Society School of Health Sciences Bangor University Bangor UK; ^2^ Social Care and Society Division of Nursing Midwifery and Social Work School of Health Sciences Faculty of Biology Medicine and Health University of Manchester Manchester UK; ^3^ School of Psychology Faculty of Health and Wellbeing University of Bolton Bolton UK; ^4^ John Spalding Library Wrexham Maelor Hospital Betsi Cadwaladr University Health Board Wrexham UK; ^5^ Division of Psychology and Mental Health Medicine and Health School of Health Sciences Faculty of Biology Medicine and Health University of Manchester Manchester UK

## Abstract

**Background:**

With a rising prison population, a substantial portion of whom are identified as substance misusers, it is important to understand the availability of treatment pathways, their successes and areas for improvement. Given the likely importance of national factors in criminal justice and substance use service provision, we chose to focus on one country.

**Aim:**

To review substance misuse treatment and outcomes for such treatments as implemented in British prisons for men.

**Methods:**

We conducted a mixed‐methods systematic review, searching Ovid MEDLINE, Ovid Embase, APA PsycINFO, CINAHL Plus, Sociology Collection, Web of Science Core Collection and Social Science Premium Collection between 1 January 2000 and 5 June 2024. Included were empirical, peer‐reviewed studies of processes and outcomes of UK male prison‐based substance misuse programmes. Primary outcomes included changes in substance use, withdrawal symptoms and experiences of interventions, whereas secondary outcomes encompassed quality of life, locus of control and mental health. Because of study design heterogeneity, meta‐analysis was not possible. Analysis followed JBI methodology with a convergent synthesis.

**Results:**

Fourteen studies were included: 8 qualitative, 5 quantitative studies of which 3 were randomised control trials (RCTs) and 1 mixed‐methods study, with a combined sample of 4037 participants engaged in opioid substitute treatment (OST) and/or psychosocial interventions. Four key themes emerged: the power of purposeful activity, strengthening support systems, bridging patient needs with treatment plans and, for those in opiate programmes, experiences and engagement with opioid substitution treatments.

**Conclusions:**

Participants articulated diverse treatment needs, highlighting the necessity of individualised and tailored reduction or maintenance plans. Treatment requires a comprehensive approach with the aim of facilitating effective social integration.

## 1 Introduction

The United Kingdom (UK) prison population stands at approximately 87,489 individuals, with England and Wales having the highest number of prisoners per capita in Western Europe (Jones and Lally 2024). Prisons, in some ways, provide a unique opportunity for treatment services, as those likely to benefit are generally held in one place and must consciously and deliberately opt out of treatment if offered. Substance use is one of the most important problems among prisoners, not least for associated ramifications, which include higher risk of suicide (Norman 2023; J. Shaw et al. 2003) and higher risk of violence within the prisons, reducing stability of establishment regimes (Her Majesty’s Prison and Probation Service (HMPPS) 2019; Independent Advisory Panel on Deaths in Custody 2017). In relation to outside‐world connections, substance misuse can disrupt relationships with family (Navabi et al. 2017), interrupt current or future employment (Magura and Marshall 2020; Sherba et al. 2018), pose health complications such as hepatitis, HIV and increased risk of stroke (Cheng et al. 2016; Sheikh Andalibi et al. 2021; Wang and Maher 2019), impair emotional regulation (Sinha 2008; Stellern et al. 2023) and trigger or exacerbate other mental health problems (T. M. Kelly and Daley 2013; Sinha 2008). There is a well‐established association between substance misuse and crime both in and out of custody (Independent Advisory Panel on Deaths in Custody 2017; Pierce et al. 2017; Masoudinia and Tamadoni 2022).

After release from prison, individuals who have misused substances also face an elevated risk of relapse (Larney et al. 2018; Masoudinia and Tamadoni 2022; Winter et al. 2019), recidivism (Fazel et al. 2017; The Centre for Social Justice 2015) and mortality (Phillips and Roberts 2019; Pratt et al. 2010), especially within the initial month after release (Cooper et al. 2023; Zlodre and Fazel 2012). Poor social support, lack of suitable prosocial accommodation, absence of a daily routine, absence of drug‐free peers, lack of family support, not having an assigned case manager or support worker and the absence of continuity of care are all key risk factors for relapse after release (Binswanger et al. 2012; Jamin et al. 2021; Joudrey et al. 2019; Van Olphen et al. 2006). Continuity of care is hard to ensure when individuals leave custody (ACMD 2019; Masoudinia and Tamadoni 2022). Support is often unavailable or engagement with it poor. Only about 12% of individuals who have a history of heroin dependency left prison with naloxone (Advisory Council on the Misuse of Drugs 2019), and only 37% identified as requiring continuity of care upon release had engaged with substance misuse treatment services within 3 weeks prior to release in 2021/2022 (Office for Health Improvement and Disparities 2023). Nearly 75% of prisoners with opioid use disorder experience a relapse within the first 3 months after release (Kinlock et al. 2008). Furthermore, 60% of all drug‐related fatalities occur within 12 weeks of release (Merrall et al. 2010), underscoring the importance of understanding substance misuse and the support systems in place during this vulnerable time.

Given the range of identified harms associated with substance misuse, further efforts to prevent or resolve the impacts are necessary. Despite over 2 decades of national recognition of shortfalls in substance misuse treatment for offenders (Black 2020; PricewaterhouseCoopers 2007; Patel 2010), to date, no systematic review has explored the substance misuse treatment experiences, processes and outcomes for men in UK prisons. As part of the background literature review, the authors conducted a systematic search of international systematic reviews, identifying 18 relevant reviews with comparable eligibility criteria. Most of these international systematic reviews included evaluations undertaken in the United States (US) (Boksán et al. 2023; Malta et al. 2019; Mitchell et al. 2012; Moore et al. 2019; Sugarman et al. 2020; Thekkumkara et al. 2022; Woodhouse et al. 2016). No reviews that are specific to the United Kingdom were identified. Differences in judicial, social, treatment and cultural contexts, therefore, limit the transferability of these findings to the United Kingdom (Boksán et al. 2023; Montanari et al. 2022).

There is a notable difference in drug use patterns between the United Kingdom and the United States; 48% of individuals in UK secure settings report heroin use compared to 23% of US state prisoners, where the opioid crisis has been largely driven by prescription drug misuse (Brinkley‐Rubinstein et al. 2018; Office for Health Improvement and Disparities 2025). UK prisons routinely provide opioid substitution therapy (OST) alongside psychosocial interventions (Independent Expert Working Group 2017), whereas OST access in the United States is limited and varies widely by state, with a reliance on nonresidential cognitive behavioural therapy (CBT), intensive residential drug abuse programmes (RDAP) and community diversion schemes such as drug courts and Law Enforcement Assisted Diversion (LEAD) (Beaton and Gerber 2025; Collins et al. 2017; Brinkley‐Rubinstein et al. 2018).

In the United Kingdom, there have been key developments in the criminal justice system in treating and managing substance use since 2000. These have included the introduction of Drug Treatment and Testing Orders (DTTOs), the Criminal Justice Interventions Programmes (CJIP) (Home Office 2004), the RAPt programme, the Counselling, Assessment, Referral, Advice and Throughcare (CARAT) programme and methadone treatment in the early 2000s (Kopak et al. 2015; Wright et al. 2014). It is on record that prior to 2000, there were no methodologically rigorous evaluations of in‐prison treatments for substance use (Harrison et al. 2003).

The aim of this review, therefore, is to explore research reported since 2000 into the experiences, processes and outcomes of treatment for substance misuse within prison settings in the United Kingdom, allowing for outcomes reported up to three months postrelease. Primary outcomes include changes in substance use, withdrawal symptoms and experiences of interventions; secondary outcomes include mechanisms such as locus of control and correlates such as changes in other aspects of mental health, quality of life (QoL) and personal and family relationships.

### 1.1 Methods

We followed the Preferred Reporting Items for Systematic reviews and Meta‐Analyses protocols (PRISMA; Moher et al. 2009; M. J. Page et al. 2021) for this review (see also Supporting Information S1, PRISMA checklist). A search of the Cochrane Database of Systematic Reviews and PROSPERO confirmed that there were no similar systematic reviews or planned reviews. We registered the protocol for this review (PROSPERO, CRD42023425189).

### 1.2 Search Strategy

Comprehensive search strategies were developed using terms for ‘substance misuse/dependence’ and prisons and treatment. The full search algorithm is reported in Supporting Information S2.

The search strategy was peer reviewed by a fellow librarian using the PRESS checklist (McGowan et al. 2016).

Seven bibliographic databases—Ovid MEDLINE, Ovid Embase, APA PsycINFO, CINAHL Plus, Sociology Collection, Web of Science Core Collection and Social Science databases—were searched (by librarian PG and KB) on 14/07/2023 and updated on 05/06/2024, using 01/01/2000 as the start date for includable publications. Published geographical search filters for the United Kingdom were modified and used for Ovid MEDLINE (Ayiku et al. 2017) and Ovid Embase (Ayiku et al. 2019). Aside from limits on dates, as described above, limits were not applied when undertaking the searches to ensure all available literature was found. Reference lists for all included studies were scrutinised for further relevant publications. We contacted study authors where details were missing (e.g., individual characteristics such as gender and age). Duplicate papers were removed in EndNote X9 before being uploaded to Covidence for screening.

### 1.3 Study Eligibility

#### 1.3.1 Types of Study

There were no limits on the study design except that included studies had to be empirical, peer‐reviewed, published in English and focusing on the experiences, processes and outcomes of UK prison‐based substance misuse treatment to include short‐term (up to 3 months) follow‐up after treatment begun in prison. Allowance for mixed methods was made to provide a more comprehensive and rich understanding of both interventions and context (see R. L. Shaw et al. 2014). For the same reasons, there was no restriction on inclusion by nature of outcomes reported.

#### 1.3.2 Inclusion Criteria

Participants had to be male and over 18 and in a UK prison at the time of the intervention.

Eligible interventions were any pharmacological intervention (e.g., buprenorphine, methadone); any psychosocial intervention (e.g., therapeutic community, group work, case management, cognitive behavioural therapy, interpersonal psychotherapy, motivational interviewing) and medicine management programmes.

#### 1.3.3 Exclusion Criteria

Being female, aged 17 years or under, in the community at the time of the intervention or in a prison outside the United Kingdom.

### 1.4 Quality Assessment

Two researchers (from KB, AK, NB and RA) independently evaluated all studies using the Joanna Briggs Institute (JBI) checklist for qualitative research (Lockwood et al. 2015), the JBI critical appraisal checklist for analytical cross‐sectional studies for quantitative research (Moola et al. 2017) and the JBI critical appraisal tool for the assessment of risk of bias for RCTs (Tufanaru et al. 2020). The mixed‐methods study was assessed using the mixed‐methods appraisal tool (MMAT) (Hong et al. 2018). Disagreements were resolved by a third reviewer.

### 1.5 Data Extraction

Cochrane guidelines were followed (Liberati et al. 2009). First, two reviewers independently screened all articles by reviewing and screening titles and abstracts (KB and RA). Secondly, the remaining full‐text articles were examined in detail and assessed for their eligibility (KB, RA and NB). Articles that did not meet the inclusion criteria were excluded. Subsequently, all references were examined to ensure no relevant articles were missed. Any disagreements were resolved by a discussion with a third reviewer (AK, PH and AO’N). One researcher extracted the data from all eligible full‐text studies and organised the variables into a piloted pro forma (KB), a modified version of the Cochrane ‘Data Collection Form: Intervention review—RCTs and non‐RCTs’. Data extraction accuracy was assessed by a second reviewer (NB), and any discrepancies were resolved by discussion with a third reviewer (AK, PH or AO’N).

### 1.6 Analysis

A meta‐analysis could not be undertaken because of the heterogeneity in the study design, sample population, age, programme duration and intensity, control group variations and different outcome measures across the 14 studies guided by Joanna Briggs Institute (JBI) methodology (The Joanna Briggs Institute 2014), a convergent synthesis design approach used to integrate data from both positivist and constructivist paradigm perspectives for all studies (Hong et al. 2017). This combines ‘*the power of stories and the power of numbers*’ (Pluye and Hong 2014, 29), enabling construction of a comprehensive picture of the experiences, processes and outcomes associated with substance misuse treatment in prison and postrelease. This integrative approach supports a deeper and complete understanding of how such treatments work, or, apparently, do not (Dixon‐Woods et al. 2004; Stern et al. 2020). Quantitative data from six studies, including three RCTs (see Supporting Information S3), were converted into narrative summaries to allow integration with the qualitative data. This process, ‘qualitisation’, involved turning statistical results into simple narrative descriptions, such as average scores or percentages. These summaries were then grouped into categories along with the qualitative data and analysed thematically (Bazeley 2012; Hong et al. 2017; Stern et al. 2020). All findings were pooled and presented in a summary table (see Table 1). Reflexive thematic analysis was used to identify key themes following Braun and Clarke's (2012) six‐step inductive approach: (1) familiarisation with the data, (2) coding, (3) generating initial themes, (4) reviewing themes, (5) defining and naming themes and (6) writing up. Two researchers (KB and AO’N) coded the data in NVivo 15, explored themes, then presented them for further refinement through discussions with the team (Braun and Clarke 2006). When interpreting the data, the researchers acknowledged their personal and professional backgrounds, experiences and positions and how these contributed to the overall generation of the themes (Braun and Clarke 2020).

**TABLE 1:** Study characteristics.

Study (author, year)	Study objective	Type of intervention (programme)	Length of treatment programme	Setting	Postrelease follow‐up	Sample (*n*, demographics)	Study design	Measures	Effectiveness of intervention (summary of outcomes)	Quality assessment
Broderick and Kouimtsidis (2007)	To investigate prisoners' perceptions of existing treatment	Detoxification Methadone Buprenorphine	Not reported	HMP Wandsworth	N/A	8 male participants Mean age, 34 years All participants were intravenous opiate users prior to entering custody The average length of drug use was 16.5 years	Qualitative (semi‐structured interviews)	N/A	All prisoners agreed that their current treatment did not meet their needs. Higher doses of methadone and buprenorphine detoxification were cited as preferred treatment options	40% high risk of bias
Brown et al. (2016)	To evaluate the Master Gardener (MG) programme at HMP Rye Hill	Master Gardener programme	Not reported	HMP Rye Hill	N/A	25 males residing on the recovery wing Age of the sample was not reported Substance use information not reported	Qualitative (focus groups)	Not reported	Engagement in the MG programme reduced substance misuse, had a positive impact on participants' health and subjective sense of well‐being	70% low risk of bias
Disbury et al. (2015)	To assess whether the programmes facilitate improvements in cognitive skill deficits associated with relapse and recidivism	Rehabilitation of Addicted Prisoners Trust (RAPt), Alcohol Dependence Treatment Programme (ADTP) Bridge Programme	6 weeks	Not reported	N/A	2299 male participants Mean age, 31.9 years ADTP: Participants reported alcohol (81.5%) as their primary substance Bridge: Participants reported cocaine (crack/powder) (35.7%) as their primary substance	Quantitative (psychometric questionnaires)	Severity of Dependence Scale (SDS) The Alcohol Use Disorders Identification Test (AUDIT) The Offender Group Reconviction Scale 3 (OGRS‐3) The Drug‐Taking Confidence Questionnaire (DTC‐Q‐8) The University of Rhode Island Change Assessment Scale (URICA) The Social Problem‐Solving Inventory‐Revised Short‐Form (SPSI‐R:SF)	There were post‐treatment improvements on measures of a range of dynamic risk factors for relapse and reoffending. Treatment completion was a fundamental tenet in the improvement of scores	90% low risk of bias
Elison et al. (2016)	To explore Breaking Free Online’s (BFO’s) potential to provide support to prisoners' substance misuse recovery and continuity of care postrelease and examine quantitative outcomes provided by prisoners who have used the programme	Breaking Free Online	1–12 weeks Mean treatment engagement, 4.58 weeks	Not reported	N/A	101 male participants Mean age, 34.8 years Participants reported heroin (27.1%) as their primary substance and alcohol (17.6%)	Mixed methods (psychometric assessments; semi‐structured interviews)	Recovery Progression Measure (RPM) The World Health Organisation Quality of Life Measure (WHOQoL‐BREF) The Severity of Dependence Scale (SDS)	There were self‐reported improvements in the participants' quality of life, and drug and alcohol dependence. No significant differences were found post‐treatment for unhelpful behaviours and ‘recovery progression’	80% low risk of bias
Garvey et al. (2021)	The aim was to compare outcomes for justice‐involved individuals who were triaged into either Breaking Free Online (BFO) or Pillars of Recovery (PoR) based on their risk of reoffending	Breaking Free Online (BFO) Pillars of Recovery (PoR)	Breaking Free Online (BFO), 8 weeks Pillars of Recovery (PoR), 12 weeks	Not reported	N/A	466 male participants (BFO—332) (PoR—134) Mean age, 35.6 years Substance use information not reported	Quantitative (data analysis)	Recovery Progression Measure (RPM) The World Health Organisation Quality of Life Measure (WHOQoL‐BREF) The Severity of Dependence Scale (SDS)	Both treatment groups showed lower levels of substance dependence, improved quality of life and lower levels of biopsychosocial impairment	50% moderate risk of bias
Harman and Paylor (2004)	To explore the efficacy of throughcare arrangements for drug users (or former drug users) leaving prison and returning to a specific locality	CARAT (Counselling, Assessment, Referral, Advice and Throughcare)	Not reported	HMP Haverigg HMP Kirkham HMP Lancaster HMP Preston HMP Wymott	6–24 days	9 male participants due for release Mean age, 29 years Participants reported heroin (89%) as their primary substance	Qualitative (semi‐structured interviews)	N/A	Six participants reported not returning to dependent drug use and all stated they had not re‐offended since release. Two participants reported having used illegal drugs (one was using cannabis regularly and the other had used heroin on one occasion). Four were recalled to prison and all relapsed while out on licence	50% moderate risk of bias
Howells et al. (2002)	Efficiency of lofexidine for opioid detoxification in a prison setting in the United Kingdom	Detoxification Lofexidine methadone	10 days	Not reported	N/A	76 participants Lofexidine: Mean age, 29.8 years; methadone: Mean age, 30.5 years Use of heroin was reported by 97.1% (n‐66) of the participants during the previous month	Quantitative (RCT)	Withdrawal Problems Scale (WPS) Short Opiate Withdrawal Scale (SOWS) The Severity of Dependence Scale (SDS)	The highest withdrawal scores in both treatment groups occurred on day 1. There were no observed significant differences in the maximum withdrawal scores between the two treatment groups	70% low risk of bias
Johnstone et al. (2011)	Subjective experiences of subutex prescribing in a male prison cohort	Detoxification Buprenorphine (subutex)	18—30 days	Not reported	N/A	14 participants who had previously undertaken a methadone detox Mean age, 31 years The use of heroin was reported by 100% of the participants	Qualitative (semi‐structured interviews)	N/A	The withdrawal discomfort for participants prescribed subutex in this study was found to be significantly less than their previous experience of withdrawal from opiates using methadone during previous custodial sentence treatment. The majority perspective was that methadone was associated with a return to illicit use	50% moderate risk of bias
G. Page et al. (2016)	Exploration of the impact of how the Drug Recovery Wings (DRWs) have shaped work with opiate‐dependent prisoners in the nine‐adult pilot DRW prisons.	Drug Recovery Wings Type A: Comprehensive clinical services Type B: Selective clinical/psychosocial support Type C: Abstinence Type D: Intensive psychosocial support	Between 20 min per month and 29 h weekly	Not reported	N/A	102 male participants (stated ‘aimed for’) Female data were not used	Qualitative (semi‐structured interviews)	N/A	*Type A*: No group programmes or interventions on building recovery capital. Several participants identified medication as the only meaningful support *Type B*: The goal of abstinence proved elusive *Type C*: Excluded heroin users *Type D*: Supported former heroin users through a full detoxification process	30% high risk of bias
Sheard et al. (2009)	Comparing dihydrocodeine and buprenorphine for opiate detoxification in a UK prison	Detoxification Dihydrocodeine Buprenorphine	20 days	HMP Leeds	1 and 3 months (6 months data excluded)	90 participants undertaking a detox (63 participants using opiates gave a urine sample at 5 days post detoxification) The use of heroin was reported by 100% of the participants	Quantitative (RCT)	N/A	At the completion of detoxification, a higher proportion of participants allocated to buprenorphine provided a urine sample negative for opiates (abstinent) compared with those who received dihydrocodeine. At the 1 and 3‐month follow‐up points, there were no significant differences for urine samples negative for opiates between the two groups	70% low risk of bias
Smith and Ferguson (2005)	To explore the social and psychological processes of individuals in prison undergoing drug rehabilitation treatment and how to manage their recovery	HM Prison Drug Rehabilitation Programme (DRP)	Not reported	Not reported	N/A	11 male participants enrolled on the DRP Aged 21–35 Between 5 and 18 years of substance abuse	Qualitative (semi‐structured interviews)	N/A	The participants reported that becoming clean from drugs involved increasing their awareness of what drove drug use and adopting strategies to avoid drug use	80% low risk of bias
Sondhi et al. (2016)	Exploring the content of the alcohol brief intervention (ABI); the process by which an ABI was delivered and prisoner/staff perceptions of what elements an effective brief intervention may comprise	Alcohol brief intervention (ABI)	Single intervention	Not reported	N/A	21 males (female data excluded) No mean age for males All interviewed reported as polydrug users	Qualitative—(focus groups)	Alcohol Use Disorders Identification Test (AUDIT)	Participants highlighted the difficulty in understanding the nature of the questions asked as part of AUDIT at this stage and stated a concern that they might be ‘judged’ by healthcare professionals	20% high risk of bias
Taylor et al. (2020)	Assess the effect of a clinical psychologist‐facilitated group intervention on male prisoners' locus of control of drinking behaviour	Clinical psychologist‐facilitated group intervention	3 weeks	Not reported	N/A	128 males	Quantitative (RCT)	The Locus of Control of Behaviour Scale (LCB) The Comprehensive Psychopathological Rating Scale (CPRS) The Beck Depression Inventory (BDI) The Stages of Change (SOC) questionnaire	There was no specific benefit of the intervention on locus of control	76% low risk of bias
Turgoose et al. (2018)	Reporting on the feasibility and outcomes of the service as a pilot intervention	Veteran Forensic Substance Misuse Service (VFSMS)	Up to 16 weeks	Not reported	Up to 3 months.	Age range, 28–39 years. Participants reported cocaine (*n =* 1) and alcohol (*n =* 1) as their primary substance	Qualitative (case studies)	Recovery Star measure Alcohol Use Disorders Identification Test (AUDIT)	The case management approach had a positive impact by liaising with the right services for that individual. All three cases described improvements in their substance misuse	40% high risk of bias

## 2 Results

### 2.1 Study Characteristics

**FIGURE 1:**
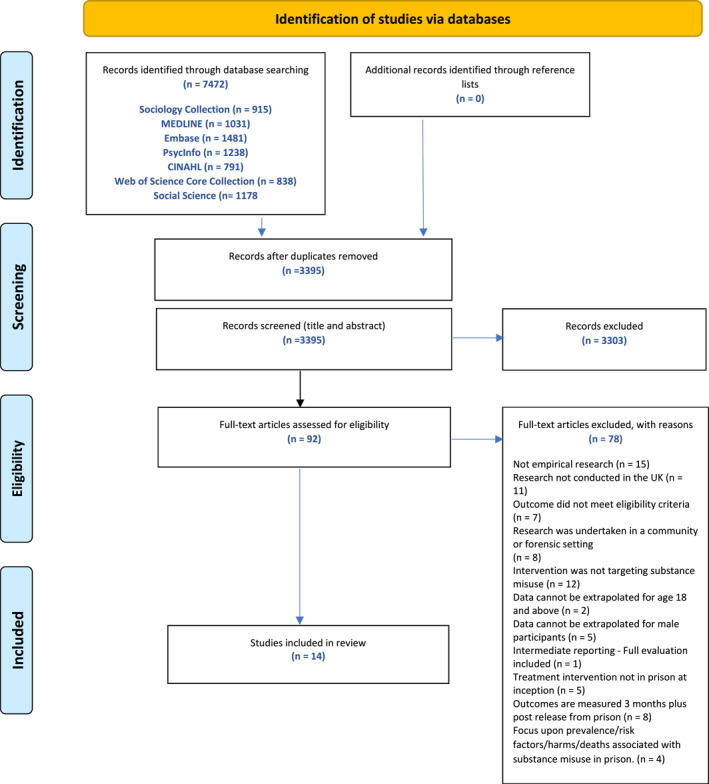
PRISMA diagram.

The database searches identified 7472 references, of which 4077 were duplicates. Thus, 3395 unique studies were screened by title and abstract, of which 92 met the eligibility criteria for full‐text screening (see Figure 1). Fourteen studies were eligible for inclusion: 8 qualitative studies, 5 quantitative studies, of which 3 were randomised controlled trials (RCTs), and one was a mixed‐methods study.

Table 1 provides an overview of all 14 included studies. Data were collected from 4037 males participating in a substance misuse treatment programme. Four of them evaluated opiate substitute treatments (OSTs) (Broderick and Kouimtsidis 2007; Howells et al. 2002; Johnstone et al. 2011; Sheard et al. 2009) and one a combination of OST and psychosocial intervention (G. Page et al. 2016). Six studies focused on psychosocial interventions (Disbury et al. 2015; Elison et al. 2016; Garvey et al. 2021; Harman and Paylor 2004; Smith and Ferguson 2005; Taylor et al. 2020) including case management (Brown et al. 2016) and one used a psychometric tool as a framework for an intervention (Sondhi et al. 2016). All included studies were published between 2002 (Howells et al. 2002) and 2021 (Garvey et al. 2021). Treatment durations ranged from a single interaction (Sondhi et al. 2016) to 3 months (Brown et al. 2016). Because of incomplete reporting across studies, particularly regarding mean age, ethnicity and substance use prior to intervention, we have presented only the characteristics and demographic information that were consistently provided across all studies (see Table 1).

### 2.2 Quality Assessment

The quality score of the 14 included studies ranged from 20% (Sondhi et al. 2016) to 90% (Disbury et al. 2015) (for summary, see Supporting Information S4). Six were assessed to be at low risk of bias (Brown et al. 2016; Disbury et al. 2015; Elison et al. 2016; Howells et al. 2002; Sheard et al. 2009; Smith and Ferguson 2005; Taylor et al. 2020), three at moderate risk of bias (Garvey et al. 2021; Harman and Paylor 2004; Johnstone et al. 2011) and four at high risk of bias (Broderick and Kouimtsidis 2007; G. Page et al. 2016; Sondhi et al. 2016; Turgoose et al. 2018). Studies with a high risk of bias often showed incongruity between the stated philosophical perspective and the research methodology and between the research methodology and the interpretation of results (Broderick and Kouimtsidis 2007; G. Page et al. 2016; Turgoose et al. 2018).

### 2.3 Synthesis of Qualitative and Quantitative Studies

#### 2.3.1 Theme 1: The Power of Purposeful Activity

Evidence from the reviewed studies suggested that purposeful activities encouraged a reduction in substance use and provided a sense of ‘meaning’ and ‘purpose’. This was described as a valuable replacement, where individuals felt it was important to engage in activities to keep their minds occupied and ‘*fill the void of time previously consumed with drug abuse*’ (Brown et al. 2016; Harman and Paylor 2004; Smith and Ferguson 2005, 66).

Some of the treatment programmes included in this study reported incorporating purposeful activities such as gardening and in‐cell workbooks (Brown et al. 2016; Harman and Paylor 2004; Taylor et al. 2020). Purposeful activities are often referred to as ‘recovery capital’. Cloud and Granfield (2008, 32) defined recovery capital as ‘*the breadth and depth of internal and external resources that can be drawn upon to initiate and sustain recovery*’. The lack of integration of these activities can contribute to low rates of abstinence or failure to reduce substance use (G. Page et al. 2016).

Engaging in a structured role although in prison, such as a garden project, not only reduced drug use but also led to reported health and mood improvements among participants. Additionally, it enhanced the ability to manage emotions, largely due to being outdoors, engaging in a purposeful activity and having the opportunity to talk with others involved in the project (Brown et al. 2016).

Having goals, particularly once back in the ‘*real world*’ (Smith and Ferguson 2005, 66), was seen as an important part of the recovery process by individuals (Brown et al. 2016; Harman and Paylor 2004; G. Page et al. 2016). Structure, including employment and other activities, was reported as fundamental upon release, as well as a focus on future planning and emphasising a positive future (Sondhi et al. 2016). Some participants were unaware of postrelease opportunities (Smith and Ferguson 2005). Assistance with employment was consistently reported as a factor in preventing relapse, providing something to engage with when entering the community, reducing loneliness and providing an income to fund constructive activities (Brown et al. 2016; Harman and Paylor 2004; G. Page et al. 2016; Smith and Ferguson 2005). One treatment intervention that included planning for release and assistance with engaging in hobbies, activities and employment found that all three case studies involved had reduced their drinking, drug use and addictive behaviours (Turgoose et al. 2018). Lack of meaningful activity was reported as difficult to cope with, creating feelings of loneliness, boredom, guilt and a sense of social isolation (Harman and Paylor 2004).

#### 2.3.2 Theme 2: Strengthening Support Systems

Participants reported needing accessible support if they were to reduce their drug use or ‘get clean’ (Brown et al. 2016; Sondhi et al. 2016; G. Page et al. 2016; Smith and Ferguson 2005; Turgoose et al. 2018), elsewhere reporting that without a treatment plan ‘*you're gonna use*’ (Smith and Ferguson 2005, 65). This was related to both custody and release. Individuals described being unaware of what opportunities exist postprison. Entering the community was reported as frightening and isolating for some, particularly if the individual had never had the responsibility of taking care of themselves drug‐free or of having had a drug‐free social structure/network (Harman and Paylor 2004; Smith and Ferguson 2005). Some individuals lacked social/familial support (Smith and Ferguson 2005). One participant described immense loneliness upon release and attributed loneliness as a motivating factor to using substances; being ‘*gouched out*’ (sedated) means you can be alone, without noticing that you do not have company (Harman and Paylor 2004, 76).

Family support or the desire to regain family connections is described as a motivating factor for achieving abstinence (Smith and Ferguson 2005). Participants also reported that support from peers, prison staff and healthcare providers is essential (Brown et al. 2016; Johnstone et al. 2011; G. Page et al. 2016; Smith and Ferguson 2005). The development of strong peer groups and close support from services was particularly linked to a greater willingness to detox (G. Page et al. 2016) and remain heroin‐free (Johnstone et al. 2011). Support upon release could assist individuals in overcoming the challenge of distancing themselves from their old lifestyles (Harman and Paylor 2004).

#### 2.3.3 Theme 3: Bridging Patient Needs and Treatment Plans

What was noticeable was the wide range of preferences among participants regarding what they wanted a substance misuse treatment programme to deliver or provide.

Upon reception into prison, participants described feeling unheard and that pharmacological interventions were decided for them, without their input into their treatment plan (Broderick and Kouimtsidis 2007). Preferences varied among individuals, with some favouring methadone maintenance (Broderick and Kouimtsidis 2007; G. Page et al. 2016), whereas others sought rapid detoxification (Broderick and Kouimtsidis 2007), with other participants preferring buprenorphine as a pharmacological intervention (Johnstone et al. 2011). It was reported that in addition to OST, participants indicated that additional support, such as counselling, would help (Broderick and Kouimtsidis 2007; G. Page et al. 2016).

Both intensive and lower‐intensity programmes yielded positive outcomes (Brown et al. 2016; Elison et al. 2016; Garvey et al. 2021; Turgoose et al. 2018). The 6‐week RAPt programme found there were positive changes in problem‐solving orientation, impulsiveness and the ability to abstain from drugs. The post‐treatment scores were higher among programme completers than noncompleters (Disbury et al. 2015) (see Supporting Information S3, Table 2). By contrast, in the RCT by Taylor et al. (2020), there was no significant difference in primary (locus of control) or secondary (e.g., depression) outcomes between completers and noncompleters; however, in the strained prison environment, only seven men completed all nine sessions (Taylor et al. 2020, 1844) (see Supporting Information S3, Table 1). The primary distinction between the two intensive programmes was the length and structure. Notably, the RAPt programme was based on a 12‐step approach, ran for twice the duration and involved full‐day group sessions, compared to the group psychological intervention, which consisted of three to four sessions per week for 3 weeks (Disbury et al. 2015; Taylor et al. 2020). Work by Sondhi et al. (2016) showed that programmes using educational, graphic images for shock value were ineffective. Specifically, alcohol brief interventions (ABIs) were criticised for focusing too much on highlighting past mistakes rather than promoting positive change and future planning. They also found that those participating in ABIs were often poly‐drug users who used alcohol alongside other substances, and none expressed a desire to reduce their alcohol consumption.

Participants viewed the Alcohol Use Disorders Identification Test (AUDIT) primarily as a statistical exercise, with mixed opinions on its usefulness in understanding their drinking levels (Sondhi et al. 2016). Some participants found it unhelpful, whereas others reported in the Breaking Free Online (BFO) programme the benefits of understanding the harmful effects of their drinking (Elison et al. 2016). Interestingly, staff delivering ABIs felt the use of AUDIT was not used as a gateway into initiating a brief intervention; rather, the AUDIT scores were held in case files. Staff felt the use of ABIs would be more effective to administer prior to release (Sondhi et al. 2016). The BFO programme findings emphasised the importance of having a programme accessible upon release for continued care, not least as individuals participating in the programme reported anxieties around postrelease (Elison et al. 2016).

The Pillars of Recovery (PoR) programme, which lasted 4 weeks longer than the BFO and included both clinical and psychosocial support, showed significantly higher quality‐of‐life rates and biopsychosocial scores than did the BFO. Substance dependence scores improved for both types of interventions, with greater improvements corresponding to the intensity of the intervention, aligning with the individual's needs (Garvey et al. 2021) (see Supporting Information S3, Table 4). Smith and Ferguson (2005) attributed the captive nature of being in prison as a motivator to engage in treatment programmes.

Findings from Johnstone et al. (2011) and Broderick and Kouimtsidis (2007) indicated that counselling or additional support for illicit drug use was seen as beneficial by participants. Reflecting on shameful drug‐using experiences proved beneficial in aiding recovery, allowing participants to discuss and acknowledge the consequences of their behaviour towards themselves, their families, friends and society (Smith and Ferguson 2005). Patterns such as depression, guilt, anger and low self‐esteem were identified as potential triggers for relapse. Managing and self‐regulating these emotions through coping strategies seems essential to abstinence (Smith and Ferguson 2005). Participants who did not receive counselling often sought support from their peers instead (Brown et al. 2016; G. Page et al. 2016). BFO participants reported feeling that prison was an unsafe environment for sharing personal information and opening up (Elison et al. 2016), whereas the use of an online computer programme bypassed these concerns effectively. In Harman and Paylor's study (2004), service users did not value ‘talking’ unless their practical needs were addressed, also a finding in the PoR and BFO programmes. Despite completing a treatment programme in custody, participants expressed anxiety about returning to their preprison lifestyle, overwhelming any benefits from programme completion (Elison et al. 2016). Individuals reported a desire to go to the gym and attend anger management sessions after release to help manage emotions (Smith and Ferguson 2005; Turgoose et al. 2018), improve their mental health (Brown et al. 2016; Turgoose et al. 2018) and participate in meaningful activities. Gaining employment and resettling were the most frequently mentioned needs, also doubling as motivators for preventing relapse (Broderick and Kouimtsidis 2007; Elison et al. 2016; Harman and Paylor 2004; Smith and Ferguson 2005; Turgoose et al. 2018).

For treatment to be truly effective, participants reported one must possess a genuine desire to change, coupled with readiness and motivation (Broderick and Kouimtsidis 2007; Harman and Paylor 2004; Johnstone et al. 2011). This intrinsic motivation not only drives the recovery process but also significantly impacts treatment retention (Johnstone et al. 2011).

#### 2.3.4 Theme 4: Experiences and Engagement With Opioid Substitution Treatments

Five papers in this review reported on the processes, perceptions or outcomes of receiving OST. Two studies focused upon the subjective experiences (Broderick and Kouimtsidis 2007; Johnstone et al. 2011), one on opioid reduction experiences as part of a wider study into DRWs (G. Page et al. 2016) and two RCTs compared outcomes: One examined abstinence between two opioid agents (Sheard et al. 2009), whereas the other compared withdrawal effects from opioid treatments (Howells et al. 2002).

No significant differences in the severity of withdrawal were found when methadone was compared to lofexidine (Howells et al. 2002). Although lofexidine is no longer prescribed as an OST in UK prisons, participants in a 10‐day RCT reported no significant differences in withdrawal symptoms between lofexidine (SD 208.3) and methadone (SD 184.4) using self‐rated withdrawal symptom scales. This may, however, have been due to differences in treatment retention between the methadone (87.5%) and lofexidine (70%) groups (see Supporting Information S3, Table 1). Although the RCT assessed the overall effectiveness in reducing opiate withdrawal symptoms, it did not report on specific individual symptoms, such as physical withdrawal symptoms, making direct comparison with the qualitative findings difficult. In those studies, methadone was described as having worse withdrawal effects than heroin (Broderick and Kouimtsidis 2007; Johnstone et al. 2011), with descriptions of methadone as making individuals feel ‘*strung out*’ and ‘*stoned*’, similar to heroin but more addictive (Johnstone et al. 2011, 58). Buprenorphine, by contrast, was preferred for its effectiveness, fewer cravings and more manageable withdrawals. Notably, participants reported increased sleep during detox on methadone (8.6 h) compared to buprenorphine (7.4 h); long‐term sleep quality improved with buprenorphine (Johnstone et al. 2011).

Buprenorphine and dihydrocodeine were compared in an RCT to determine the preferred agent for achieving abstinence (Sheard et al. 2009). Importantly, dihydrocodeine is no longer prescribed as an OST in the UK prison system. This study found buprenorphine more effective in achieving abstinence from illicit opiates 5 days post‐treatment, though both agents had identical outcomes 3 months postrelease, with a 25% abstinence rate, although reflecting only two of eight participants. This was one of only two studies that followed participants after release. Harman and Paylor's (2004) study, however, had a shorter follow‐up period (six to 9 days), lack of OST engagement data and reliance on self‐reports. Sheard et al. (2009) also highlighted discrepancies between self‐reported abstinence and urine screens, with only 25% showing negative results despite 65% reporting abstinence. The limited data make it difficult to generalise OST's role in achieving abstinence, subjective preferences for it.

Aside from one participant who preferred a rapid reduction (G. Page et al. 2016), individuals described feeling dissatisfied that their OST plans did not meet their needs. Enforced OST reduction or coercion towards abstinence was reported as ineffective (Broderick and Kouimtsidis 2007; Johnstone et al. 2011) and deterred individuals from entering the DRW (G. Page et al. 2016). The DRWs excluded OST recipients unless they were willing to undergo rapid reduction, limiting those on more than 40 mg of methadone or 2 mg of buprenorphine. Individuals preferred the stability of a maintenance prescription; a methadone detoxification induced anxiety and contributed towards the scarcity of abstinence (Broderick and Kouimtsidis 2007; Johnstone et al. 2011; G. Page et al. 2016).

A further potential barrier to entering a treatment programme may stem from the uncertainty and mistrust of prison and healthcare professionals. ‘*Clients on methadone and Subutex aren't* … *motivated. [They] just want to come in, get their two mils a day, sit in their cells, not go to work, not get employment, nothing*’ (Staff, G. Page et al. 2016, 53). Participants were aware of the stigma associated with their OST and substance use, and this was further exacerbated by self‐stigma (Johnstone et al. 2011; G. Page et al. 2016).

### 2.4 Discussion

This review highlights the wide‐ranging needs of individuals using substances. Key findings highlighted the importance of individual agency in shaping personalised treatment plans, whether through psychosocial interventions or OST. Notably, participants expressed a clear preference for receiving additional support alongside their OST, indicating that integrated care approaches were more favourable. Engagement in meaningful activities, both during and after custody, was a preferable need. Such activities were seen not only as a means of filling the void left by substance use but also as essential preparation for reintegration into life beyond custody.

Understanding an individual's social background before substance misuse is vital, not least as social disadvantage can markedly increase the risk of substance use (Social Exclusion Unit 2002; Home Office 2004). Drug use can be both a consequence and a cause of social exclusion (DeWall and Pond 2011; Wesselmann and Parris 2021), subsequently leaving the individual marginalised or ‘othered’ from mainstream society (Buchanan 2004). Participants spoke to the stigma surrounding substance misuse (Johnstone et al. 2011; G. Page et al. 2016; Sondhi et al. 2016; Smith and Ferguson 2005), which has been identified as creating barriers to healthcare engagement and, consequently, having a detrimental effect on recovery (Van Boekel et al. 2013; Corrigan et al. 2009; Crapanzano et al. 2018; Mak et al. 2017; Muncan et al. 2020; Radcliffe and Stevens 2008).

This type of prejudice can lead to discrimination, manifesting as lost opportunities (employment, housing, education), coercion (by local authorities and enforced treatment programmes) and segregation (in drug wings, housing estates and prisons). Social identification theory (Tajfel and Turner 1979) explains how such marginalisation can push individuals to become more entrenched with drug‐using groups (Bourgois 1995; R. Stephens and Levine 1971; R. C. Stephens 1991). Individuals in this review emphasised the necessity of establishing a new support network by fostering non‐drug‐using connections within the community. Participants highlighted the value of integrating productive and meaningful activities, both in custody and upon release, and the known barriers to this. By building recovery capital, this process can begin in prison as a way to counteract the reported boredom, frustration and monotonous routines (O’Connor et al. 2020). This involves not only creating supportive relationships but also navigating obstacles to successful reintegration (Buchanan 2004; Cloud and Granfield 2008; HM Govt, 2010; Klingemann et al. 2001; Neale and Stevenson 2015; G. Page et al. 2016).

Participants engaging with OST emphasised the importance of combining psychosocial interventions with OST, a combination supported by previous research for improving outcomes (Amato et al. 2004, 2005; Mattick et al. 2003). The psychosocial and psychological programmes that demonstrated significant improvements were those of longer duration, requiring greater levels of participant engagement, offering more intensive content and case management support (Brown et al. 2016; Disbury et al. 2015; Garvey et al. 2021). Similar findings were reported in some of the international reviews, concluding that the most effective prison‐based interventions for reducing recidivism and drug use were longer‐term programmes addressing multiple aspects of substance misuse (Holloway et al. 2006; Mitchell et al. 2012; Woodhouse et al. 2016) or those offering intensive case management (Moore et al. 2019).

An international systematic review reported positive outcomes from ABIs, showing an increase in abstinence upon follow‐up (Newbury‐Birch et al. 2018). However, in this review, Sondhi et al. (2016) found that ABIs were not routinely initiated after AUDIT screenings. A further UK study highlighted similar concerns; although many prisoners expressed a desire for further alcohol support, services often failed to provide it, unlike for those identified as using drugs. Nearly one‐third of prisoners had alcohol problems without concurrent drug use, and three‐quarters of those with co‐occurring issues met dependency criteria for alcohol only. ABIs could present valuable opportunities for intervention, provided services are better tailored and resourced to address both alcohol and drug misuse (Kissell et al. 2014).

Although standard OST approaches have yielded mixed effectiveness, longer‐term detoxification (30+ days) shows better management of withdrawals and higher retention rates, though it is less effective in preventing relapse (Amato et al. 2005, 2013). Despite wider support for shorter detoxification plans (Amass et al. 2004; Kakko et al. 2003), only one study in this review had these findings (Broderick and Kouimtsidis 2007). Therefore, it is essential to facilitate individuals in prison who are motivated to pursue either a maintenance plan or detoxification, paying particular attention to the transition into the community. Follow‐up data were notably limited in this review. Two studies that examined individuals transitioning back into the community reported relatively high relapse rates, with the qualitative studies reporting significant challenges during this transition (Harman and Paylor 2004; Sheard et al. 2009). Although providing valuable context, the outcomes cannot be conclusively generalised. Further research specifically addressing community re‐entry is needed to provide more robust and generalisable findings.

Understanding what ‘recovery’ means to each individual feels pertinent to building recovery capital, particularly as participants described their preferred treatment approaches, given a choice (Broderick and Kouimtsidis 2007; Brown et al. 2016; Elison et al. 2016; Harman and Paylor 2004; Johnstone et al. 2011; Sondhi et al. 2016; Smith and Ferguson 2005; Turgoose et al. 2018). Tailoring interventions to meet these needs has been highlighted in several national reports in response to the government drug strategies (Black 2020; HM Inspectorate of Prisons 2014; Patel 2010; PricewaterhouseCoopers 2007; Wakeling and Lynch 2020). Reports recommend increasing funding, ensuring continuity of care from prison to community, expanding the number of qualified drug treatment staff, fostering multiagency partnerships and implementing evidence‐based services. Implementing services from a psychosocial perspective is a critical initial step, necessitating individualised and comprehensive support, including considerations for resettlement, education, training, employment, healthcare and benefits (Social Exclusion Unit 2002; Kothari et al. 2002), all while removing stigma (Black 2020). ‘*The structures, systems, processes, and relationships within institutions*’ (Best et al. 2021, 209) would need to foster a stigma‐free environment, with a strong emphasis on staff training and development.

The studies in this review were undertaken before the introduction of the most recent Prison Drugs Strategy (Her Majesty’s Prison and Probation Service (HMPPS) 2019) and the government's 10‐year strategy, *From Harm to Hope* (HM Government 2021). It is important to note that these strategies will likely be replaced with the new administration's policies. This review is particularly timely, aligning with the election of the New Labour government, critical issues such as overcrowded prisons, and the pressing need to tackle substance misuse within the criminal justice system.

The review builds on existing knowledge but emphasises the importance of revisiting and updating recommendations for future action, considering the evolving political and policy landscape.

### 2.5 Recommendations

The implications of this for policymakers are clear: Individuals should be actively involved in shaping their treatment pathways in order to improve their chances of recovery. Further, there should be an early, practical vision of next steps—both for the remaining period in prison and, at the least, over the period of transition back to the wider community. As this review has highlighted, the concept of recovery holds different meanings for different individuals, and this diversity must be considered when engaging people in substance misuse treatment programmes. A wider range of treatment options that may be joined together in different ways according to mutually assessed individual needs seems optimal.

Specifically, practical planning for transition into the community is critical. Access to safe, secure accommodation and the development of non‐substance‐using social networks are essential components of this process. Increasing the availability of community recovery centres (CRCs) (Office for Health Improvement and Disparities 2025b) that provide psychosocial support, counselling, family‐based interventions, employment and training opportunities, direct access or referral pathways to accommodation and promoting networks outside drug‐using environments can improve psychological well‐being and quality of life (J. F. Kelly et al. 2020, 2021).

National educational programmes involving prison and substance misuse professionals in all prisons are required to remove the stigma attached to substance misuse, possibly drawing upon the resources of already established antistigma campaigns, such as the *Stigma Kills campaign* (NHS addictions provide alliance, n).

### 2.6 Limitations

The limitations of our review are mainly reflective of the limitations of research in this field. Most sample sizes are small, and most of the studies included are solely qualitative. These are significant for hypothesis formation but not good at establishing what really works. Further, where outcomes have been assessed optimally—by RCT—effectiveness was only assessed immediately post‐treatment, leaving the long‐term impacts of the programmes uncertain. Interventions that are effective in the short term—particularly with substance use per se as a direct outcome—may have a fading impact, unless, perhaps, ‘top‐up’ sessions are then offered. Interventions that are apparently not immediately effective, perhaps especially when targeting some relevant personal trait, may start to show an advantage later. Above all, interventions to be offered in prison can only be appropriately tested in prison, but restrictions in the prison environment, generally beyond the control even of the prison staff, not only affect the intervention delivery, the men receiving it and the possibility of successful community carryover, but also the nature and quality of the research into them.

A more direct limitation of this review is that grey literature was excluded, potentially overlooking valuable nonacademic published research (see Lloyd et al. 2017), and, anyway, database searches may have missed some relevant studies (see Wright et al. 2011). Despite attempts to contact study authors for individual data, we could not always do so. Finally, although a thematic synthesis is useful for better understanding of the all‐important context of substance use and its treatment, it may obscure the shortcomings of individual studies (Lucas et al. 2007).

## 3 Conclusions

There remains a significant gap between recognition of the importance of substance use in offending serious enough to leave the user in prison and research measuring both the processes and outcomes of substance misuse treatment programmes. More quantitative work is required, but in formulating further programmes and research, it is crucial to gather up‐to‐date data on what individuals indicate they need for their recovery. The research reviewed here suggests that this must include empowering individuals to be involved in shaping their treatment and recovery pathways.

This study adds to existing national audits of prisons. Collectively, our findings show that treatment of substance use requires a comprehensive approach beyond opiate substitution therapy and/or early psychosocial interventions to include case management, support with housing, education, employment, benefits and meaningful activities, as well as tackling stigma. The question remains as follows: Why is this comprehensive approach not being implemented? Any short‐term costs would surely be outweighed by longer‐term gains?

## Conflicts of Interest

The authors declare no conflicts of interest.

## Supporting information


Supporting Information S1

Supporting Information S2

Supporting Information S3

Supporting Information S4

## Data Availability

The data that support the findings of this study are available from the corresponding author upon reasonable request.
