# Supplementary material for: A Systematic Review of Substance Misuse Treatment Processes and Outcomes as Implemented in Prisons for Men in the UK
Source: Crim Behav Ment Health. 2025 Aug 16;35(5):270–89. doi: 10.1002/cbm.70008 (PMC12574697; doi:10.1002/cbm.70008)
Supplement: Supplementary file 2 — Supporting Information S2 [file CBM-35-270-s003.docx]

**Supplementary Material 2 – Full Search Strategy**

**Ovid MEDLINE(R) ALL <1946 to July 13, 2023>**

1 exp Substance-Related Disorders/

2 (drug addiction or substance addiction or narcotic addiction or substance related disorder* or narcotic related disorder* or drug related disorder* or "substance use*" or "drug use*" or "narcotic use*" or substance abuse* or drug abuse* or narcotic abuse* or substance dependence* or drug dependence* or narcotic dependence* or substance misuse or drug misuse or narcotic misuse or alcohol*).ti,ab,kf.

3 1 or 2

4 Prisons/

5 Prisoners/

6 (prison* or inmate* or incarcerat* or custod* or jail* or penal or penitentiar* or detention or correctional or imprisoned or detained or detainee* or behind bars or justice system* or offender* or confinement or carceral or decarceration).ti,ab,kf.

7 4 or 5 or 6

8 exp United Kingdom/

9 (national health service* or nhs* or HMP or HMPS).ti,ab,in.

10 (english not ((published or publication* or translat* or written or language* or speak* or literature or citation*) adj5 english)).ti,ab.

11 (gb or "g.b." or britain* or (british* not "british columbia") or uk or "u.k." or united kingdom* or (england* not "new england") or northern ireland* or northern irish* or scotland* or scottish* or ((wales or "south wales") not "new south wales") or welsh*).ti,ab,jw,in.

12 or/8-11

13 (exp africa/ or exp americas/ or exp antarctic regions/ or exp arctic regions/ or exp asia/ or exp australia/ or exp oceania/) not (exp United Kingdom/ or europe/)

14 12 not 13

15 3 and 7 and 14

16 limit 15 to yr="2000 -Current"

**Ovid Embase <1974 to 2023 July 13>**

1 exp drug dependence/

2 (drug addiction or substance addiction or narcotic addiction or substance related disorder* or narcotic related disorder* or drug related disorder* or "substance use*" or "drug use*" or "narcotic use*" or substance abuse* or drug abuse* or narcotic abuse* or substance dependence* or drug dependence* or narcotic dependence* or substance misuse or drug misuse or narcotic misuse or alcohol*).ti,ab,kf.

3 1 or 2

4 correctional facility/

5 prisoner/

6 (prison* or inmate* or incarcerat* or custod* or jail* or penal or penitentiar* or detention or correctional or imprisoned or detained or detainee* or behind bars or justice system* or offender* or confinement or carceral or decarceration).ti,ab,kf.

7 4 or 5 or 6

8 United Kingdom/

9 (national health service* or nhs* or HMP or HMPS).ti,ab,in.

10 (english not ((published or publication* or translat* or written or language* or speak* or literature or citation*) adj5 english)).ti,ab.

11 (gb or "g.b." or britain* or (british* not "british columbia") or uk or "u.k." or united kingdom* or (england* not "new england") or northern ireland* or northern irish* or scotland* or scottish* or ((wales or "south wales") not "new south wales") or welsh*).ti,ab,jw,in.

12 8 or 9 or 10 or 11

13 (exp africa/ or exp americas/ or exp antarctic regions/ or exp arctic regions/ or exp asia/ or exp australia/ or exp oceania/) not (exp United Kingdom/ or europe/)

14 12 not 13

15 3 and 7 and 14

16 limit 15 to yr="2000 -Current"

**APA PsycInfo <1806 to July Week 2 2023>**

1 exp "substance use disorder"/ or exp "substance use treatment"/

2 (drug addiction or substance addiction or narcotic addiction or substance related disorder* or narcotic related disorder* or drug related disorder* or "substance use*" or "drug use*" or "narcotic use*" or substance abuse* or drug abuse* or narcotic abuse* or substance dependence* or drug dependence* or narcotic dependence* or substance misuse or drug misuse or narcotic misuse or alcohol*).ti,ab,hw.

3 1 or 2

4 Prisons/

5 incarcerated/

6 (prison* or inmate* or incarcerat* or custod* or jail* or penal or penitentiar* or detention or correctional or imprisoned or detained or detainee* or behind bars or justice system* or offender* or confinement or carceral or decarceration).ti,ab,hw.

7 4 or 5 or 6

8 (Britain or Scotland or Wales or England or Ireland).lo.

9 (national health service* or nhs* or HMP or HMPS or english or gb or "g.b." or britain* or (british* not "british columbia") or uk or "u.k." or united kingdom* or (england* not "new england") or northern ireland* or northern irish* or scotland* or scottish* or ((wales or "south wales") not "new south wales") or welsh*).ab,hw,in,ti,jx.

10 8 or 9

11 3 and 7 and 10

12 limit 11 to yr="2000 -Current"

**Sociology Collection (Proquest – includes Applied Social Sciences Index & Abstracts (ASSIA), Sociological Abstracts, Sociology Database).**

([STRICT] (noft(("drug addiction" OR "substance addiction" OR "narcotic addiction" OR "substance related disorder*" OR "narcotic related disorder*" OR "drug related disorder*" OR "substance use*" OR "drug use*" OR "narcotic use*" OR "substance abuse*" OR "drug abuse*" OR "narcotic abuse*" OR "substance dependence*" OR "drug dependence*" OR "narcotic dependence*" OR "substance misuse" OR "drug misuse" OR "narcotic misuse" OR alcohol*)) OR (MAINSUBJECT.EXACT("Drug Addiction") OR MAINSUBJECT.EXACT("Alcohol Abuse") OR MAINSUBJECT.EXACT("Substance Abuse") OR MAINSUBJECT.EXACT("Drug Abuse"))) AND (noft((Prison* OR Inmate* OR Incarcerat* OR Custod* OR Jail* OR penal OR penitentiar* OR detention OR correctional OR imprisoned OR detained OR detainee* OR "behind bars" OR "justice system" OR offender* OR confinement OR carceral OR decarcertation)) OR (MAINSUBJECT.EXACT("Imprisonment") OR MAINSUBJECT.EXACT("Prison Culture") OR MAINSUBJECT.EXACT("Offenders") OR MAINSUBJECT.EXACT("Drug Offenders") OR MAINSUBJECT.EXACT("Prisoners") OR MAINSUBJECT.EXACT("Prisons"))) AND (location((NHS OR "national health service" OR HMP OR HMPS OR english OR gb OR "g.b." OR britain* OR british* OR uk OR "u.k." OR "united kingdom*" OR england* OR "northern ireland*" OR "northern irish*" OR scotland* OR scottish* OR wales OR welsh*)) OR title((NHS OR "national health service" OR HMP OR HMPS OR english OR (gb OR "g.b." OR britain* OR (british* NOT "british columbia") OR uk OR "u.k." OR "united kingdom*" OR (england* NOT "new england") OR "northern ireland*" OR "northern irish*" OR scotland* OR scottish* OR ((wales OR "south wales") NOT "new south wales") OR welsh*))) OR abstract((NHS OR "national health service" OR HMP OR HMPS OR english OR (gb OR "g.b." OR britain* OR (british* NOT "british columbia") OR uk OR "u.k." OR "united kingdom*" OR (england* NOT "new england") OR "northern ireland*" OR "northern irish*" OR scotland* OR scottish* OR ((wales OR "south wales") NOT "new south wales") OR welsh*))))) AND pd(20000101-20231231)

**Database - CINAHL Plus with Full Text**

S1 (MH "Substance Use Disorders+")

S2 TI ( "drug addiction" OR "substance addiction" OR "narcotic addiction" OR "substance related disorder*" OR "narcotic related disorder*" OR "drug related disorder*" OR "substance use*" OR "drug use*" OR "narcotic use*" OR "substance abuse*" OR "drug abuse*" OR "narcotic abuse*" OR "substance dependence*" OR "drug dependence*" OR "narcotic dependence*" OR "substance misuse" OR "drug misuse" OR "narcotic misuse" OR alcohol* ) OR AB ( "drug addiction" OR "substance addiction" OR "narcotic addiction" OR "substance related disorder*" OR "narcotic related disorder*" OR "drug related disorder*" OR "substance use*" OR "drug use*" OR "narcotic use*" OR "substance abuse*" OR "drug abuse*" OR "narcotic abuse*" OR "substance dependence*" OR "drug dependence*" OR "narcotic dependence*" OR "substance misuse" OR "drug misuse" OR "narcotic misuse" OR alcohol* )

S3 S1 OR S2

S4 (MH "Prisoners") OR (MH "Correctional Facilities")

S5 TI ( Prison* OR Inmate* OR Incarcerat* OR Custod* OR Jail* OR penal OR penitentiar* OR detention OR correctional OR imprisoned OR detained OR detainee* OR "behind bars" OR "justice system" OR offender* OR confinement OR carceral OR decarcertation ) OR AB ( Prison* OR Inmate* OR Incarcerat* OR Custod* OR Jail* OR penal OR penitentiar* OR detention OR correctional OR imprisoned OR detained OR detainee* OR "behind bars" OR "justice system" OR offender* OR confinement OR carceral OR decarcertation )

S6 S4 OR S5

S7 (MH "United Kingdom+")

S8 TI ( (NHS OR "national health service" OR HMP OR HMPS OR english OR (gb OR "g.b." OR britain* OR (british* NOT "british columbia") OR uk OR "u.k." OR "united kingdom*" OR (england* NOT "new england") OR "northern ireland*" OR "northern irish*" OR scotland* OR scottish* OR ((wales OR "south wales") NOT "new south wales") OR welsh*) ) OR AB ( (NHS OR "national health service" OR HMP OR HMPS OR english OR (gb OR "g.b." OR britain* OR (british* NOT "british columbia") OR uk OR "u.k." OR "united kingdom*" OR (england* NOT "new england") OR "northern ireland*" OR "northern irish*" OR scotland* OR scottish* OR ((wales OR "south wales") NOT "new south wales") OR welsh*) ) OR AF ( (NHS OR "national health service" OR HMP OR HMPS OR english OR (gb OR "g.b." OR britain* OR (british* NOT "british columbia") OR uk OR "u.k." OR "united kingdom*" OR (england* NOT "new england") OR "northern ireland*" OR "northern irish*" OR scotland* OR scottish* OR ((wales OR "south wales") NOT "new south wales") OR welsh*) )

S9 S7 OR S8

S10 S3 AND S6 AND S9

S11 S3 AND S6 AND S9

Limiters - Published Date: 20000101-20231231

**Database: Web of Science Core Collection**

((TS=("drug addiction" OR "substance addiction" OR "narcotic addiction" OR "substance related disorder*" OR "narcotic related disorder*" OR "drug related disorder*" OR "substance use*" OR "drug use*" OR "narcotic use*" OR "substance abuse*" OR "drug abuse*" OR "narcotic abuse*" OR "substance dependence*" OR "drug dependence*" OR "narcotic dependence*" OR "substance misuse" OR "drug misuse" OR "narcotic misuse" OR alcohol* )) AND TS=(Prison* OR Inmate* OR Incarcerat* OR Custod* OR Jail* OR penal OR penitentiar* OR detention OR correctional OR imprisoned OR detained OR detainee* OR "behind bars" OR "justice system" OR offender* OR confinement OR carceral OR decarcertation )) AND TS=(NHS OR "national health service" OR HMP OR HMPS OR english OR (gb OR "g.b." OR britain* OR (british* NOT "british columbia") OR uk OR "u.k." OR "united kingdom*" OR (england* NOT "new england") OR "northern ireland*" OR "northern irish*" OR scotland* OR scottish* OR ((wales OR "south wales") NOT "new south wales") OR welsh*))

Timespan: 2000-01-01 to 2023-07-14

**Social Science Database**

([STRICT] (NHS OR "national health service" OR HMP OR HMPS OR english OR gb OR "g.b." OR britain* OR british* OR uk OR "u.k." OR "united kingdom*" OR england* OR "northern ireland*" OR "northern irish*" OR scotland* OR scottish* OR wales OR welsh*)) AND ((prison* OR inmate* OR incarcerat* OR custod* OR jail* OR penal OR penitentiar* OR detention OR correctional OR imprisoned OR detained OR detainee* OR behind bars OR justice system* OR offender* OR confinement OR carceral OR decarceration) AND PEER(yes)) AND (([STRICT] (drug addiction OR substance addiction OR narcotic addiction OR substance related disorder* OR narcotic related disorder* OR drug related disorder* OR "substance use*" OR "drug use*" OR "narcotic use*" OR substance abuse* OR drug abuse* OR narcotic abuse* OR substance dependence* OR drug dependence* OR narcotic dependence* OR substance misuse OR drug misuse OR narcotic misuse OR alcohol*)) AND PEER(yes))
