# Supplementary material for: A Systematic Review of Substance Misuse Treatment Processes and Outcomes as Implemented in Prisons for Men in the UK
Source: Crim Behav Ment Health. 2025 Aug 16;35(5):270–89. doi: 10.1002/cbm.70008 (PMC12574697; doi:10.1002/cbm.70008)
Supplement: Supplementary file 3 — Supporting Information S3 [file CBM-35-270-s002.docx]

**Supplementary Material 3 – Quantitative Data Tables**

**Table 1. Summary of Randomised Controlled Trial Results**

| **Study** *(author, year)* | **Measure, timepoint** | **Group 1 no. participants with outcome data** | **Group 1, mean (SD)** | **Group 2, no. participants with outcome data** | **Group 2, mean (SD)** | **95% confidence interval/Relative risk** | **P-value** |
| --- | --- | --- | --- | --- | --- | --- | --- |
| Howells et al., 2002 | Withdrawal Problems Scale (WPS)  Short Opiate Withdrawal Scale (SOWS)  10 days | Lofexidine Group | 596.1 (208.3) | Methadone Group | 572.1 (184.4) | Not reported | 0.632 |
|  |  | 32 |  | 36 |  |  |  |
| Sheard et al., 2009 | Urine Screening  5 days  1 month  3 months | Dihydrocodeine Group | Not reported | Buprenorphine  Group | Not reported | - 1. 0.90, 1.54)   1.0 (0.27, 3.72)  1.0 (0.59, 1.68) | 0.43  Not reported  1.00 |
|  |  | 48  5  4 |  | 42  10  8 |  |  |  |
| Taylor et al, 2020 | Locus of control (LOC)  Beck Depression Scale global score | Intervention Group | 33.7 (11.7)  17.9 (11.2) | Control Group | 31.9 (11.8)  16.0 (9.9) | - 1.7 ( -5.1 to 1.6)  -1.8 (-5.5 to 1.8) | 0.329  0.324 |
|  |  | 68  68 |  | 60  60 |  |  |  |

**Table 2. OLS Regression Results Predicting Post-Treatment Psychometric Scores (Disbury et al., 2015)**

| **Variable** | **DTCQ–DR b (SE)** | **DTCQ–ALC b (SE)** | **SPSI–PPO b (SE)** | **SPSI–NPO b (SE)** |
| --- | --- | --- | --- | --- |
| Constant | 46.90 (4.48)** | 50.89 (4.16)** | 35.80 (1.59)** | 28.82 (1.53)** |
| Pretest score | .31 (.02)** | .25 (.02)** | .49 (.02)** | .62 (.02)** |
| Age | .21 (.07)** | .16 (.06)** | -.12 (.01)** | .11 (.01)** |
| Race/ethnicity: Asian | 1.10 (2.75) | -.25 (2.63) | .73 (.43) | -.67 (.37) |
| Race/ethnicity: Black | -1.91 (1.60) | 2.22 (1.58) | -.40 (.26) | -.40 (.22) |
| Race/ethnicity: Other | .33 (2.23) | -2.28 (2.15) | .54 (.34) | .01 (.30) |
| Primary substance: Cocaine | -1.96 (1.86) | .08 (1.82) | .13 (.29) | -.26 (.26) |
| Primary substance: Opiates | -3.74 (2.08) | .59 (2.07) | -.33 (.33) | -.59 (.29)* |
| Primary substance: Other | -2.13 (1.93) | .22 (1.88) | .23 (.30) | -.28 (.27) |
| Secondary substance used | -1.81 (1.54) | -1.53 (1.36) | -.22 (.22) | -.04 (.19) |
| Dependence severity | -.06 (.06) | -.11 (.06)* | .00 (.01) | -.01 (.01) |
| OGRS score | .02 (.03) | .01 (.03) | .00 (.00) | -.00 (.00) |
| Index offense: Drug | -1.40 (1.74) | -.34 (1.73) | -.14 (.28) | .26 (.24) |
| Index offense: Theft & kindred | -3.44 (1.38)** | -1.04 (1.33) | -.25 (.21) | -.25 (.19) |
| Index offense: Other | -1.14 (1.62) | -.33 (1.52) | -.14 (.24) | -.30 (.22) |
| ADTP program participation | -.98 (1.58) | 2.54 (1.52) | –.08 (.24) | .15 (.22) |
| Completion | 10.66 (2.33)** | 12.41 (2.21)** | .60 (.36) | .74 (.32)* |
| Model R², F | 0.19, 20.04** | 0.15, 17.30** | 0.45, 77.45** | 0.60, 141.02** |
| n | 1,400 | 1,552 | 1,525 | 1,525 |

*Note.* DTCQ = Drug-Taking Confidence Questionnaire; SPSI = Social Problem-Solving Inventory; PPO = Positive Problem Orientation; NPO = Negative Problem Orientation; OGRS = Offender Group Reconviction Scale; ADTP = Alcohol Dependence Treatment Programme.*p<.01 **p<.01.

**Table 3. Self-Reported Psychometric and Alcohol and Drug Outcomes Among Service Users (Elison et al., 2015)**

| **Outcome Measure** | **Mean (SD) Baseline** | **Mean (SD) Post-Treatment** | **df** | **F / t** | **p-value** | **Effect Size (r)** |
| --- | --- | --- | --- | --- | --- | --- |
| WHOQoL-BREF | 10.52 (4.43) | 12.31 (4.80) | 84 | 3.488 | <0.0001 | 0.38 |
| SDS – Alcohol | 5.83 (4.39) | 4.21 (4.55) | 84 | −2.486 | 0.013 | 0.57 |
| SDS – Drugs | 6.68 (6.50) | 5.80 (4.83) | 84 | −4.744 | <0.0001 | 0.36 |
| Alcohol Consumption | 117.34 (111.63) | 17.49 (57.71) | 84 | −5.054 | <0.0001 | 0.74 |
| Drug Use | 89.14 (256.09) | 47.10 (194.52) | 84 | −6.186 | <0.0001 | 0.67 |
| RPM – Total | 37.47 (14.26) | 35.62 (16.26) | 84 | −0.815 | 0.415 | 0.01 |
| RPM – Difficult Situations | 5.48 (2.95) | 4.88 (3.06) | 84 | −2.237 | 0.025 | 0.24 |
| RPM – Negative Thoughts | 5.60 (3.12) | 4.72 (3.08) | 84 | −3.143 | 0.002 | 0.34 |
| RPM – Physical Sensations | 4.87 (2.91) | 4.27 (2.87) | 84 | −2.452 | 0.014 | 0.27 |
| RPM – Unhelpful Behaviours | 5.21 (3.27) | 5.05 (3.34) | 84 | −0.459 | 0.646 | 0.05 |
| RPM – Lifestyle | 5.23 (3.30) | 5.04 (3.28) | 84 | −0.455 | 0.645 | 0.06 |
| RPM – Emotions | 5.68 (2.64) | 4.89 (2.93) | 84 | −2.873 | 0.004 | 0.31 |

*Note.* RPM = Recovery Progression Measure; WHOQoL- Brief = World Health Organization Quality of Life Assessment. SDS= The severity of Dependence Scale

**Table 4. Baseline vs. Post-Treatment Comparison (Garvey et al., 2021)**

| **Group** | **Measure** | **N** | **Pre Mean (SD)** | **Post Mean (SD)** | **Pre Median** | **Post Median** | **p-value** |
| --- | --- | --- | --- | --- | --- | --- | --- |
| BFO | WHOQoL | 168 | 10.5 (4.5) | 12.7 (4.2) | 11.0 | 13.0 | p < .001 |
| BFO | SDS | 139 | 9.4 (4.0) | 6.0 (4.4) | 10.0 | 6.0 | p < .001 |
| BFO | RRPM | 169 | 31.8 (16.2) | 28.3 (16.5) | 34.0 | 30.0 | p = .61 |
| PoR | WHOQoL | 79 | 14.5 (4.8) | 19.9 (2.3) | 16.0 | 20.0 | p < .001 |
| PoR | SDS | 65 | 9.8 (4.4) | 7.3 (4.9) | 11.0 | 8.0 | p < .001 |
| PoR | RRPM | 70 | 42.2 (12.7) | 29.4 (14.8) | 45.0 | 28.0 | p < .001 |

*Note.* BFO – Breaking Free Online; POF – Pillars of Recovery; RRPM = Rapid Recovery Progression Measure; SDS = Substance Dependence Scale; WHOQoL = World Health Organization Quality of Life Assessment.
