# Supplementary material for: A Systematic Review of Substance Misuse Treatment Processes and Outcomes as Implemented in Prisons for Men in the UK
Source: Crim Behav Ment Health. 2025 Aug 16;35(5):270–89. doi: 10.1002/cbm.70008 (PMC12574697; doi:10.1002/cbm.70008)
Supplement: Supplementary file 4 — Supporting Information S4 [file CBM-35-270-s001.docx]

**Supplementary Material 4 – Quality Assessment Tables**

**Table 5 - JBI Qualitative Research Quality Assessment of Included Studies**

| **Author/Study** | **Q1** | **Q2** | **Q3** | **Q4** | **Q5** | **Q6** | **Q7** | **Q8** | **Q9** | **Q10** | **Total Score** |
| --- | --- | --- | --- | --- | --- | --- | --- | --- | --- | --- | --- |
| Broderick & Kouimtsidis, 2007 | No | Yes | No | No | No | No | No | Yes | Yes | Yes | **4** |
| Brown et al., 2016 | Yes | Yes | Yes | Yes | Yes | No | No | Yes | No | Yes | **7** |
| Harman K & Paylor, 2004 | No | Yes | Yes | No | Yes | No | No | Yes | No | Yes | **5** |
| Johnstone et al., 2011 | Yes | Yes | Unclear | Yes | Unclear | No | No | Unclear | Yes | Yes | **5** |
| Page et al, 2016 | Unclear | Unclear | Yes | Yes | No | No | No | Yes | Unclear | Yes | **3** |
| Smith & Ferguson, 2005 | Yes | Yes | Yes | Yes | Yes | Yes | No | Yes | Unclear | Yes | **8** |
| Sondhi et al., 2016 | No | Unclear | Unclear | Unclear | No | No | No | Yes | Yes | No | **2** |
| Turgoose et al., 2018 | Unclear | Yes | Yes | No | No | No | No | N/A | Yes | Yes | **4** |

1.Is there congruity between the stated philosophical perspective and the research methodology? 2. Is there congruity between the research methodology and the research question or objectives? 3. Is there congruity between the research methodology and the methods used to collect data? 4. Is there congruity between the research methodology and the representation and analysis of data? 5. Is there congruity between the research methodology and the interpretation of results? 6. Is there a statement locating the researcher culturally or theoretically? 7. Is the influence of the researcher on the research, and vice- versa, addressed? 8. Are participants, and their voices, adequately represented? 9. Is the research ethical according to current criteria or, for recent studies, and is there evidence of ethical approval by an appropriate body? 10. Do the conclusions drawn in the research report flow from the analysis, or interpretation, of the data?

**Table 6 - JBI Cross Sectional Quality Assessment of Included Studies**

| **Author/Title** | **Q1** | **Q2** | **Q3** | **Q4** | **Q5** | **Q6** | **Q7** | **Q8** | **Total Score** |
| --- | --- | --- | --- | --- | --- | --- | --- | --- | --- |
| Didsbury et al., 2015 | Yes | Yes | Yes | Yes | Yes | Unclear | Yes | Yes | 9 |
| Garvey et al., 2021 | No | Yes | Yes | Yes | No | No | Yes | Yes | 7 |

1. Were the criteria for inclusion in the sample clearly defined? 2. Were the study subjects and the setting described in detail? 3. Was the exposure measured in a valid and reliable way? 4. Were objective, standard criteria used for measurement of the condition? 5. Were confounding factors identified? 6. Were strategies to deal with confounding factors stated? 7. Were the outcomes measured in a valid and reliable way? 8. Was appropriate statistical analysis used?

**Table 7 - JBI RCT Quality Assessment** **of Included Studies**

| **Author/Title** | **Q1** | **Q2** | **Q3** | **Q4** | **Q5** | **Q6** | **Q7** | **Q8** | **Q9** | **Q10** | **Q11** | **Q12** | **Q13** | **Total Score** |
| --- | --- | --- | --- | --- | --- | --- | --- | --- | --- | --- | --- | --- | --- | --- |
| Howells et al., 2002 | Yes | Yes | No | Yes | Yes | No | Yes | Yes | Unclear | No | Yes | Yes | Yes | 9 |
| Sheard et al., 2009 | Yes | No | Yes | No | No | Yes | No | Yes | Yes | Yes | Yes | Yes | Yes | 9 |
| Taylor et al., 2020 | Unclear | No | Unclear | Unclear | Unclear | Unclear | No | Yes | Yes | Yes | Yes | Yes | Yes | 6 |

**Bias related to selection and allocation** 1. Was true randomization used for assignment of participants to treatment groups? 2. Was allocation to treatment groups concealed? 3. Were treatment groups similar at the baseline? **Bias related to administration of intervention/exposure** 4. Were participants blind to treatment assignment? 5. Were those delivering the treatment blind to treatment assignment? 6. Were treatment groups treated identically other than the intervention of interest?  **Bias related to assessment, detection and measurement of the outcome** 7. Were outcome assessors blind to treatment assignment? 8. Were outcomes measured in the same way for treatment groups? 9. Were outcomes measured in a reliable way? **Bias related to participant retention** 10. Was follow up complete and if not, were differences between groups in terms of their follow up adequately described and analysed? **Statistical Conclusion Validity** 11. Were participants analysed in the groups to which they were randomized? 12. Was appropriate statistical analysis used? 13. Was the trial design appropriate and any deviations from the standard RCT design (individual randomization, parallel groups) accounted for in the conduct and analysis of the trial?

**Table 8 - MMAT Quality Assessment** **of Included Study**

| **Author/s** | **Screening Questions** | | **Qualitative Studies** | | | | | **Quantitative Descriptive Studies** | | | | | **Mixed Methods Studies** | | | | | **Total Score** |
| --- | --- | --- | --- | --- | --- | --- | --- | --- | --- | --- | --- | --- | --- | --- | --- | --- | --- | --- |
|  | **S1.** | **S.2** | **1.1** | **1.2** | **1.3** | **1.4** | **1.5** | **4.1** | **4.2** | **4.3** | **4.4** | **4.5** | **5.1** | **5.2** | **5.3** | **5.4** | **5.5** | 80% or 4****  High Quality |
| Elison et al., 2016 | Yes | Yes | Yes | Yes | Yes | Can’t tell | Yes | Yes | Yes | Yes | No | Yes | Yes | Yes | Yes | Can’t tell | Yes |  |

**Screening Questions** S1. Are there clear research questions? S2. Do the collected data allow the research questions to be addressed? 1. **Qualitative Studies** 1.1. Is the qualitative approach appropriate to answer the research question? 1.2. Are the qualitative data collection methods adequate to address the research question? 1.3. Are the findings adequately derived from the data? 1.4. Is the interpretation of results sufficiently substantiated by data? 1.5. Is there coherence between qualitative data sources, collection, analysis, and interpretation? 4. **Quantitative Descriptive Studies** 4.1. Is the sampling strategy relevant to address the research question? 4.2. Is the sample representative of the target population? 4.3. Are the measurements appropriate? 4.4. Is the risk of nonresponse bias low? 4.5. Is the statistical analysis appropriate to answer the research question? 5. **Mixed Method Studies** 5.1. Is there an adequate rationale for using a mixed methods design to address the research question? 5.2. Are the different components of the study effectively integrated to answer the research question? 5.3. Are the outputs of the integration of qualitative and quantitative components adequately interpreted? 5.4. Are divergences and inconsistencies between quantitative and qualitative results adequately addressed? 5.5. Do the different components of the study adhere to the quality criteria of each tradition of the methods involved?
